# Early warning scoring systems versus standard observations charts for wards in South Africa: a cluster randomized controlled trial

**DOI:** 10.1186/s13063-015-0624-2

**Published:** 2015-03-20

**Authors:** Una Kyriacos, Jennifer Jelsma, Michael James, Sue Jordan

**Affiliations:** Division of Nursing and Midwifery, Department of Health and Rehabilitation Sciences, Faculty of Health Sciences, University of Cape Town, Anzio Road, Cape Town, 7925 South Africa; Department of Health and Rehabilitation Sciences, Faculty of Health Sciences, University of Cape Town, Anzio Road, Cape Town, 7925 South Africa; Department of Anaesthesiology, Groote Schuur Hospital/University of Cape Town, Anzio Road, Cape Town, 7925 South Africa; School of Human and Health Sciences, Swansea University, Singleton Park, Swansea, Wales SA2 8PP UK

**Keywords:** Cluster randomized trial, Developing country, In-service training, Monitoring (physiological), Pragmatic randomized controlled trial, Vital signs, Scoring systems (MeSH checked 3 April 2013)

## Abstract

**Background:**

On South African public hospital wards, observation charts do not incorporate early warning scoring (EWS) systems to inform nurses when to summon assistance. The aim of this trial was to test the impact of a new chart incorporating a modified EWS (MEWS) system and a linked training program on nurses’ responses to clinical deterioration (primary outcome). Secondary outcomes were: numbers of patients with vital signs recordings in the first eight postoperative hours; number of times each vital sign was recorded; and nurses’ knowledge.

**Methods/design:**

A pragmatic, parallel-group, cluster randomized, controlled clinical trial of intervention versus standard care was conducted in three intervention and three control adult surgical wards in an 867-bed public hospital in Cape Town, between March and July 2010; thereafter the MEWS chart was withdrawn. A total of 50 out of 122 nurses in full-time employment participated. From 1,427 case notes, 114 were selected by randomization for assessment.

The MEWS chart was implemented in intervention wards. Control wards delivered standard care, without training. Case notes were reviewed two weeks after the trial’s completion. Knowledge was assessed in both trial arms by blinded independent marking of written tests before and after training of nurses in intervention wards. Analyses were undertaken with IBM SPSS software on an intention-to-treat basis.

**Results:**

Patients in trial arms were similar. Introduction of the MEWS was not associated with statistically significant changes in responses to clinical deterioration (50 of 57 received no assistance versus 55 of 57, odds ratio (OR): 0.26, 95% confidence interval (CI): 0.05 to 1.31), despite improvement in nurses’ knowledge in intervention wards. More patients in intervention than control wards had recordings of respiratory rate (27 of 57 versus 2 of 57, OR: 24.75, 95% CI: 5.5 to 111.3) and recordings of all seven parameters (5 of 57 versus 0 of 57 patients, risk estimate: 1.10, 95% CI: 1.01 to 1.2).

**Conclusions:**

A MEWS chart and training program enhanced recording of respiratory rate and of all parameters, and nurses’ knowledge, but not nurses’ responses to patients who triggered the MEWS reporting algorithm.

**Trial registration:**

This trial was registered with the Pan African Clinical Trials Registry (identifier: PACTR201309000626545) on 9 September 2013.

**Electronic supplementary material:**

The online version of this article (doi:10.1186/s13063-015-0624-2) contains supplementary material, which is available to authorized users.

## Background

Critically ill patients are increasingly being nursed on general wards, where it is reported that monitoring of vital signs is infrequent and inadequate [1,2]. Interpretation of signs of clinical deterioration is poor, and responses to clinical deterioration are inappropriate [3]. Clinical and physiological deterioration, including changes in respiratory rates, occurs six to eight hours [4] before cardiopulmonary arrest. Arrest often occurs after a period of unrecognized, slow, and progressive physiological deterioration [5].

Multi-professional in-hospital courses for life support after a catastrophic event are well established [6]. However, there are few examples of training programs for early recognition and management of adult patients with impending critical illness. One such program, the ‘Acute Life-threatening Events - Recognition and Treatment’ (ALERT) course [6], is based on the assumption of pre-existing knowledge of the biosciences, but previous research [7] suggests that this underpinning knowledge may be suboptimal. Traditional assumptions that mortality and determinants of survival fall within the domain of medical care may be inhibiting nurse-led research in this area, but there is increasing evidence that these outcomes are ‘nursing sensitive’ [8] .

Introducing an early warning scoring (EWS) or modified early warning scoring (MEWS) system is complex [9]. However, observational studies [4,10] and before and after evaluation studies [11] indicate that EWS systems improve detection of clinical deterioration. This paper describes a pragmatic, parallel-group, cluster randomized, controlled trial evaluating the impact of a MEWS training program and published consensus-derived MEWS observations chart (Additional file 1: Figure S1) [12,13] on the proportion of patients with physiological variables recorded and nurses’ responses.

To our knowledge, there are no published randomized controlled trials or other experimental studies on the implementation and evaluation of MEWS training programs and recording systems. The purpose of this trial of intervention versus standard care was to test the impact of a MEWS system and a linked training program.

## Methods

### Design

We designed a prospective, pragmatic, cluster randomized trial with two arms (intervention versus no intervention), using hospital surgical wards as the unit of randomization, testing the hypothesis that nurse training plus introduction of a locally developed MEWS chart would improve knowledge, recording of vital signs, and nurses’ responses to predetermined physiological thresholds. Randomization was at two levels: wards and patient records. Our study satisfied the six criteria for pragmatic trials [14]. There were no deviations from the protocol.

A pragmatic, cluster randomized trial design was chosen to minimize contamination, as nurses might use information for all patients, consciously or subconsciously [15]. A permanent nurse was unlikely to be assigned to both an intervention and control ward, therefore allocation of all nurses in respective clusters to the intervention or control arm, rather than individual nurses, limited the threat of contamination. Secondly, monitoring, recording, and interpreting patients’ vital signs and responding to abnormal physiology is the responsibility of the ward nursing team, and evaluation of the quality of such care is best assessed at ward or cluster level. The Consolidated Standards of Reporting Trials (CONSORT) guidelines [14,16-18] were used to report the study (Additional file 2).

### Study sites

The study was conducted between 1 March and 31 July 2010 in six purposively sampled adult wards for general, vascular, and orthopedic surgery in an 867-bed academic public (government) hospital in Cape Town, South Africa. The traditional ‘cardiac arrest team’ , comprising ICU nurses and doctors, had been replaced by ward response teams more than two decades previously. There was no hospital-wide emergency response system for predefined thresholds for deterioration in physiological variables and no EWS system in place on any general wards. Approval for implementation of the MEWS chart was limited to the study period; usual care was resumed at the conclusion of the study.

### Sampling

Patient case notes from intervention and control wards provided clinical and demographic data.

#### Inclusion criteria for records from intervention and control wards

All patients identified from case notes who were aged 14 years or more [19], had had a general anaesthetic, and were admitted to one of the six research wards between 1 May and 31 July 2010, were eligible for inclusion.

#### Exclusion criteria for patient records

Those with absent, incomplete or unavailable records (for example due to medico-legal review) were excluded from the study. Incomplete records were defined as not including observations charts and patient progress notes. If either was absent, case notes were discarded, and replaced by an alternative random number. Patients designated ‘not for resuscitation’ in their case notes were excluded from the study as this might have affected nurses’ responses to changes in vital signs. Any case notes of patients transferred to high dependency care from the operating room, or from the ward, within eight hours of their operation (including patients who had died there) were excluded from the study because the study focus was ward-based vital sign monitoring.

#### Sample size determination for record review

We are unaware of any randomized controlled trials examining quality of recording of postoperative vital signs on general wards, or nurses’ responses to abnormal physiology, as defined by MEWS scores; this might be attributed to the relative novelty of this method of monitoring vital signs. However, the United Kingdom Health Foundation’s Safer Patients Initiative (SPI) controlled before and after study [20] indicates that a multi-component organizational intervention increases respiratory rate monitoring frequency from 40 (93 out of 233) to 69% (165 out of 239) in control hospitals, and from 37 (141 out of 381) to 78% (296 out of 379) in intervention hospitals. Preparatory work [13] indicated that patients’ respiratory rate was the parameter monitored least frequently (1.8%, one out of 55) in these wards. We felt an increase in respiratory rate monitoring frequency from 1.8 (one out of 55) to at least 20.0% (11 out of 55)[21], would be clinically important and, based on the results of the study above, achievable.

A sample of 114 records (57 from each trial arm) was calculated to be sufficient to detect a difference of 18.2% (from 1.8 to 20.0%) between arms in the frequency of monitoring respiratory rates, with 80% power and 5% significance [22]. No information was available on the impact of clustering on MEWS system use, and no intra-cluster correlation coefficient could be calculated.

All nurses who were permanently employed full time (professional, staff, and auxiliary) on the six study wards, and covering day and night shifts, were invited to participate in the training evaluation (n = 62 on intervention wards, n = 60 on control wards). Part-time staff were temporary and recruited from agencies. These individuals fluctuated daily, so they could not be included. Student nurses were excluded as they were allocated on a temporary, *ad hoc* basis. Unlike agency staff, permanent nurses were assigned to single wards, reducing the threat of contamination.

#### Randomization and sequence generation of wards

Six wards were randomized to either the intervention or control trial arms by the drawing of sealed lots [23].

#### Randomization and sequence generation of patient records

Following conclusion of the intervention, patients’ records were obtained for review. To select a total of 114 records, 19 records were randomly selected from all six wards. For patients who met the inclusion criteria, random numbers were generated using SPSS (IBM Corp., IBM SPSS Statistics for Windows, Version 19.0, Armonk, NY): each record was screened and, if incomplete, unavailable, or meeting other exclusion criteria, an alternative random number was generated and a new record was selected by further randomization. Records of all patients who met inclusion criteria, including those who died on the ward, were examined.

#### Recruitment of nurses

Post-randomization, all nurses in the intervention arm were approached to seek voluntary participation in the training program (two declined). Nurses in the control arm were recruited for voluntary participation in the pre- and post-intervention knowledge testing only (none declined) (Additional file: 3 Pre- and post- intervention test).

### Allocation concealment and blinding

Allocation concealment was managed by an independent person (administrator, NL) drawing sealed lots, being blinded to the ward names on the lots and to outcome (intervention and control trial arms).

Patients were unaware of their allocation. Nurses who participated voluntarily in the training program and knowledge testing were not blinded to allocation, as the purpose of the training was to implement the MEWS chart.

Data collectors (JO and TW) were blinded to the allocation and implementation of the intervention. Data analysts for the knowledge tests (JO) and case notes (JJ and SJ) were blinded to the codes identifying trial arms. The first author (UK) was the Principal Investigator (PI) and was not blinded for two reasons: 1) the PI conducted nurses’ knowledge tests and trained nurses in the intervention wards in the use of the MEWS chart; and 2) the hospital database and operating room register identified patients by name and folder number, and both were searched manually for inclusion criteria for participants. Once identified, the PI requested case notes by name and folder number from the Records Department and signed a confidentiality clause. The CONSORT extended pragmatic checklist allows for an explanation of the absence of blinding.

### Interventions

#### The Cape Town modified early warning score training program

The Cape Town MEWS training program and manual [21] included basic physiology and literature on the advantages of using patients’ early warning scores as part of the handover process, thereby focusing shift handover meetings on patient safety [24]. Three experts validated the content and face validity of the knowledge test, training manual, and power point presentation, using a checklist and scale of one to four to establish the Index of Content Validity (CVI) [25], giving a rating of three to four (high content-validity) for each. Recommended changes were made before administration.

Attendance at the two-hour interactive training sessions was dependent on staffing and workload as there were no resources to replace nurses on training. Wards released one to three nurses at a time, so five repeat training sessions were needed for day staff. Nurses on night duty were trained individually or in pairs when they were next on day duty. Nurses’ knowledge pre-and post-intervention was tested in all six wards (Additional file 3). Of the 11 official languages, English is used for recordings in patient notes, and was the medium of instruction and testing, although it is a second or third language for most nurses.

Staggered pre-intervention knowledge testing was conducted between 12 March and 23 March for nurses in both trial arms. The MEWS training program was conducted between 12 March and 22 April. The MEWS chart was introduced on 1 May and removed at midnight on 31 July. Post-intervention knowledge testing was conducted between 3 August and 13 August.

#### Implementing the Cape Town modified early warning score observation chart

While standard care (existing observations chart [12]) continued in three control wards, the Cape Town MEWS observations chart [12] (Additional file 1: Figure S1) was implemented in the three intervention wards. This MEWS chart indicates which scores should be reported, the degree of urgency needed, and where a total score should be entered.

During training, nurses were instructed to assign the correct score for each vital sign recorded, total the scores, and respond as follows: for a single parameter MEWS of one, to recheck the measurement after half an hour and report if there was no improvement; for a single parameter MEWS of two, to recheck the measurement after five minutes and report immediately if there was no improvement; for a critical single parameter MEWS of three, to report urgently; and for a total MEWS of three or higher, to report urgently. Senior ward nurses who had received training were recruited as MEWS Project Leaders, identifiable by a lanyard bearing a card with this title. Their task was to keep the project on track, order sufficient copies of the chart from the first author, and to ensure that new staff received training. Training manuals were available on all intervention wards. Patients’ records (n = 114) were reviewed two weeks after the completion of the study, on 31 July 2010. Data were captured electronically on an Excel criterion-based record review form. Researchers recoded vital signs from existing observations charts onto MEWS charts for all patients in the control arm and patients in the intervention arm where the MEWS charts were blank (36.8%, 21 out of 57). Data were examined for nurses’ responses to signs of disturbed physiology by reviewing patient progress notes for recorded interventions (above).

#### Primary outcome measure

The primary outcome measure was nurses’ documented responses to abnormal vital signs measures, as determined by rechecking vital signs or calling for assistance from a more senior nurse or medical doctor, for patients who met the pre-selected criteria for the intervention (above).

#### Secondary outcome measures

Secondary outcome measures were:

1. the number of patients with physiological variables (respiratory and heart rate, oxygen saturation, systolic blood pressure, temperature, level of consciousness, and urine output) recorded on the MEWS chart in intervention wards and on existing vital signs charts in control wards in the first eight postoperative hours;
2. the number of times each vital sign was recorded in the first eight postoperative hours in both trial arms; and
3. pre- and post-intervention test scores of nurses’ knowledge by blinded independent marking.

### Data analysis

Statistical analyses were undertaken with IBM SPSS Statistics version 19 on an intention-to-treat basis [26]. A per protocol analysis was undertaken [27] as a secondary sensitivity analysis [28]. Records from the intervention arm where the MEWS was not used were excluded in the per protocol analysis. Tests of normality for distribution of data followed the convention of using the Shapiro-Wilk test for a sample size smaller than 50 [29], and we used the Kolmogorov-Smirnov test for a sample size greater than 50.

Bivariate associations were explored by comparisons of means or ranks (t tests, Mann-Whitney U, or equivalents for paired data), as appropriate. The χ^2^ test was used with Yates’ continuity correction for 2 × 2 tables. Fisher’s exact test was substituted for low numbers. Haldane’s correction was taken to calculate a risk estimate, when odds ratios could not be calculated. Statistical significance was set at *P* <0.05. Low numbers in outcome variables precluded adjusted analyses.

### Ethical considerations

The study was approved by the University of Cape Town, Faculty of Health Sciences Human Research Ethics Committee (HREC REC reference 192/2009), the South African National Clinical Trial Registry (SANCTR DOH 27-0713-4483), and the Pan African Clinical Trial Registry (PACTR201309000626545), hospital management, and clinical structures. The South African National Health Research Ethics Committee (NHREC) was notified of the study in 2009 (application 2009/3483). The study was conducted according to the principles of the Declaration of Helsinki [30].

Written informed consent was obtained from nurse participants on intervention wards for the training program and knowledge testing, and on control wards for knowledge testing only [31]. Cluster randomized controlled trials raise important ethical issues in relation to the nature and practice of informed consent [15,32]. When the MEWS observations chart was implemented on the three intervention wards, consent was not obtained from individual nurses [33] or patients, as the institutional consent (guardianship) [34] was considered to be the ‘community consent’ [15] on behalf of individuals in the intervention clusters. The University of Cape Town, Faculty of Health Sciences Human Research Ethics Committee waived the need for written informed consent from individual patients whose records were reviewed. In terms of South African legislation at that time (National Health Act No. 61 of 2003, Section 16 (2)) [35] a health care provider may examine a user’s health records for the purposes of research without authorization if the research will not obtain information relating to the identity of the user.

## Results

### Record review

**Figure 1:**
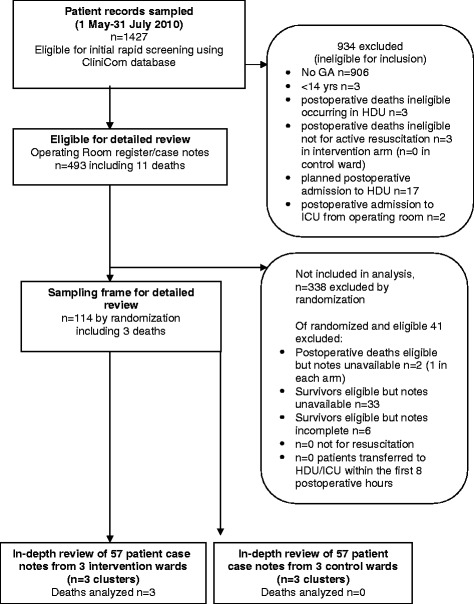
**Flow diagram of criterion-based record review process of trial.** Note on figure: All records were sampled from the six research wards. CliniCom database, the electronic hospital information system; GA, general anaesthetic; HDU, high dependency unit; ICU, intensive care unit.

Of 1,427 patients undergoing surgery between 1 May and 31 July 2010 (Figure 1), 493 were aged 14 years or more and had received a general anaesthetic. The 1,427 included 11 patients who had died during this time (n = 8 in the intervention arm; n = 3 in the control arm). Of these 11, five had received a general anaesthetic, but records were unavailable for two of the five. The other six records were ineligible for analysis because three deaths occurred outside the wards and three patients who died were ‘not for resuscitation’. This left three records for analysis, all of which were from the same ward in the intervention arm. Figure 1 details the selection of records. Amongst the 482 survivors, 33 records were unavailable, six were incomplete, 0 patients were ‘not for resuscitation’ , and 0 patients were transferred to a high dependency unit (HDU) or ICU within eight hours of operation.

#### Baseline demographic and clinical characteristics of patients

Baseline demographic data and clinical characteristics of patients are presented in Tables 1 and 2, indicating that patients in trial arms were similar. The difference in proportion of patients undergoing different surgery reflected the wards’ surgical specialties (Table 1).

**Table 1:** **Type of surgery in intervention and control arms**

	**Intervention arm (n = 57)**	**Control arm (n = 57)**
**Type of surgery**	**Number (%)**	**Number (%)**
General	16 (28.1)	19 (33.3)
Vascular	2 (3.5)	6 (10.5)
Gastrointestinal	1 (1.8)	13 (22.8)
Orthopedic	38 (66.7)	19 (33.3)

**Table 2:** **Baseline demographics and clinical characteristics of patients in intervention and control arms**

**Characteristic**	**Intervention arm**	**Control arm**
	**(n = 57)**	**(n = 57)**
	**Number (%)**	**Number (%)**
Sex: female	21 (36.8)	36 (63.2)
Pre-existing comorbidity:		
Myocardial infarction	0	3 (5.3)
Renal	1 (1.8)	0
Diabetes mellitus	13 (22.8)	4 (7.01)
Carcinoma	4 (7.01)	10 (17.5)
Respiratory	9 (15.8)	9 (15.8)
Cerebrovascular Accident	5 (8.8)	7 (12.3)
Hypertension	17 (29.8)	19 (33.3)
1 co-morbidity	19 (33.3)	26 (45.6)
2 co-morbidities	17 (29.8)	13 (22.8)
3 co-morbidities	10 (17.5)	6 (7.0)
4 co-morbidities	2 (3.5)	0
Age in years:		
Median	49.00	45.00
Range	14-76	14-84
Interquartile range	29	26

Marginally more patients in the intervention group (48 out of 57, 84.2%) had up to four comorbidities than those in the control group (45 out of 57, 78.9%).

#### Nurses’ responses to high and low threshold modified early warning scores for vital sign recordings (intention-to-treat analysis)

Nurses had used the MEWS chart as a single parameter tracking tool; no-one had calculated a total MEWS for each observation time-point despite training. The numbers of abnormal vital signs recordings, summarized in Table 3, were similar in trial arms. Responses to MEWS triggers that should have been reported for patients who met pre-selected criteria for intervention are summarized in Table 4. Nurses in the intervention arm did not record responses to 121 out of 128 (94.5%) MEWS triggers that should have been reported for 50 out of 57 (87.7%) patients. Nurses in the control arm failed to record responses to 91 out of 93 (97.8%) deranged physiological parameters recoded into MEWS triggers that should have been reported for 55 out of 57 (96.5%) patients. These differences in response rates between the two trial arms did not reach statistical significance (odds ratio (OR): 2.63, 95% confidence interval (CI): 0.53 to 12.97) or the number of patients affected in each trial arm (OR: 0.26, 95% CI: 0.05 to 1.31). Overall, nurses in both trial arms reported nine out of 221 (4.1%) deranged physiological parameters for nine out of 105 (8.6%) patients who needed to be assessed. Nurses did not report two abnormal signs (respiratory rate, systolic blood pressure (BP), and/or heart rate) respectively for two of the three patients who died; the other patient had two out of two abnormal signs (heart rate and temperature) reported.

**Table 3:** **Number of abnormal vital signs recorded per patient (intention-to-treat analysis)**

**Number of abnormal vital signs**	**Intervention arm**	**%**	**Control arm**	**%**
	**n**		**n**	
0	9	15.79	12	21.05
1	19	33.33	26	45.61
2	17^a^	29.82	13	22.81
3	10	17.54	6	10.53
4	2	3.51	0	
Total	57	100	57	100

^a^Two patients died.

No significant differences identified, df = 4, χ^2^ 5.05, *P* = 0.28. df, the degrees of freedom, or the number of ‘entities’ that are free to vary when a statistical test is applied; this also determines the probability distribution used for the test statistic.

**Table 4:** **Nurses’ responses to disturbed physiology (MEW score 1 to 3)** ^**a**^ **: numbers of patients that should have triggered and did trigger responses in their first eight postoperative hours**

	**Intervention arm n = 50 patients**			**Control arm n = 55 patients**		
**Parameter and MEW scores**	**Number of MEWS trigger points**	**Response to number of patients**		**Number of MEWS trigger points**	**Response to number of patients**	
		**Yes (%)**	**No (%)**		**Yes (%)**	**No (%)**
Respiratory rate MEWS						
1	15	0	15 (100)	0	0	0
2	6	0	6 (100)	0	0	0
3	0	0	0	0	0	0
Total	21	0	21 (100)	0	0	0
Heart rate MEWS						
1	11	0	11 (100)	13	0	13 (100)
2	7	1 (14.3)	6 (85.7)	6	0	6 (100)
3	1	0	1 (100)	0	0	0
Total	19	1 (5.3)	18 (94.7)	19	0	19 (100)
Oxygen saturation MEWS						
1	0	0	0	1	0	1 (100)
2	1	0	1 (100)	0	0	0
3	0	0	0	0	0	0
Total	1	0	1 (100)	1	0	1 (100)
Systolic blood pressure MEWS						
1	11	1 (9.1)	10 (90.9)	20	0	20 (100)
2	7	0	7 (100)	4	0	4 (100)
3	7	2 (28.6)	5 (71.4)	3	1 (33.3)	2 (66.7)
Total	25	3 (12.0)	22 (88.0)	27	1 (3.7)	26 (96.3)
Temperature MEWS						
1	22	2 (9.1)	20 (90.9)	18	0	18 (100)
2	7	0	7 (100)	4	0	4 (100)
3	0	0	0	1	0	1 (100
Total	29	2 (6.9)	27 (93.1)	23	0	23 (100)
Level of consciousness MEWS						
1	9	0	9 (100)	7	0	7 (100)
2	0	0	0	0	0	0
3	0	0	0	0	0	0
Total	9	0	9 (100)	7	0	7 (100)
Urine output MEWS						
1	11	0	11 (100)	5	0	5 (100)
2	6	0	6 (100)	4	0	4 (100)
3	7	1 (14.3)	6 (85.7)	7	1 (14.3)	6 (85.7)
Total	24	1 (4.2)	23 (95.8)	16	1 (6.3)	15 (93.7)
Overall total, primary outcome	128	7 (5.5)	121 (94.5)	93	2 (2.2)	91 (97.8)

Intention-to-treat analysis.

^a^No distinction is made between lower and upper modified early warning score (MEWS) trigger points.

0 indicates no recordings.

Excluding level of consciousness and urine output, which should be interpreted with caution, 46 patients (80.7%) in the intervention arm (n = 57) had one to three parameters with abnormal MEWS: 19 (33.3%) patients had one abnormal parameter; 17 (29.8%) had two abnormal parameters (including the three patients who died); and 10 (17.5%) had three abnormal parameters. Two (3.5%) patients had four abnormal parameters. Nine (15.8%) patients in the intervention arm had no abnormal parameters.

In the control arm (n = 57), 45 (78.9%) patients had one to three parameters with abnormal MEWS: 26 (45.6%) patients had one abnormal parameter; 13 (22.8%) had two abnormal parameters; and six (10.5%) had three abnormal parameters. Twelve (21.0%) patients in the control arm had no abnormal parameters.

#### Patients with recorded postoperative parameters by trial arm

The MEWS chart was intended for all patients in the intervention arm but was only used with 63.2% (36 out of 57) patients. The numbers of patients with vital sign recordings within the first eight hours following surgery are shown in Table 5. All patients had recordings for blood pressure and heart rate. Patients in the intervention arm were more likely to have respiratory rate recorded (OR: 24.8, 95% CI: 5.50 to 111.32), and all seven vital signs recorded (Haldane’s estimator OR: 12.05, 95% CI: 0.650 to 223.19, *P* = 0.02).

**Table 5:** **Numbers of patients with postoperative parameter recordings by trial arm (intention-to-treat analysis)**

**Parameter**	**Intervention arm N = 57 patients**	**Control arm N = 57 patients**	***P*** **value**	**OR**	**95% CI**
	**Number (%)**	**Number (%)**			
Respiratory rate recorded	27 (47.4)	2 (3.5)	<0.001	24.75	5.50-111.33
Heart rate recorded	57 (100)	57 (100)			
Oxygen saturation recorded	7 (12.3)	2 (3.5)	0.08	3.85	0.76-19.41
Systolic blood pressure recorded	57 (100)	57 (100)	1.00		
Temperature recorded	55 (96.5)	54 (94.7)	1.00	1.53	0.25-9.51
Level of consciousness^a^ recorded	45 (78.9)	37 (64.9)	0.09	2.03	0.88-4.68
Urine output recorded	49 (86.0)	51 (89.5)	0.57	0.72	0.23-2.23
All vital signs recorded	5 (8.4)	0^b^ (0.0)	0.06	RE^b^ 1.10	1.01-1.2
Incomplete recording of all vital signs	52 (91.2)	57 (100)			
MEWS trigger should have been reported and was reported, primary outcome	7 (12.3)	2 (3.5)	0.08	0.26	0.05-1.31
MEWS trigger should have been reported and was *not* reported	50 (87.7)	55 (96.5)			

Note on table:

^a^LOC was recorded on the MEWS chart as alert (A), responds to voice (V), responds to pain (P), or unresponsive (U). On the existing chart LOC was recorded in patient progress notes as the state of wakefulness (for example ‘drowsy’) on return to ward and not Glasgow Coma Scale assessment, and this applied to 21 out of 57 (36.8%) patients in the intervention wards who did not have the MEWS chart. LOC recordings on the existing charts should be interpreted with caution.

^b^If Haldane’s estimator is used to calculate OR this circumvents zero values in cells by adding one half to each cell and gives OR = 12.05 (95% CI: 0.65 to 223.19, *P* = 0.022). CI, confidence interval; LOC, level of consciousness; MEWS, modified early warning score; OR, odds ratio; RE, risk estimate.

#### Per protocol analysis of nurses’ responses to high and low threshold MEWS vital sign recordings

Per protocol analysis (Table 6) of nurses’ responses to MEWS triggers for 63.2% (36 out of 57) of patients in the intervention arm who had the MEWS chart showed that they did not record responses to 85 out of 88 (96.6%) MEWS triggers that should have been reported. Nurses in the control ward failed to record responses to 89 out of 90 (98.9%) deranged physiological parameters recoded into MEWS triggers that should have been reported for 57 patients. Only systolic BP recordings triggered reporting: three of 15 (20.0%) abnormal recordings using the MEWS and one of 25 (4.0%) recordings using the standard chart. These differences did not reach statistical significance. Overall, nurses in both trial arms reported a combined total of four out of 178 (2.2%) deranged physiological parameters for four out of 93 (4.3%) patients who needed to be assessed. Patients with the MEWS chart in the intervention arm were more likely to have recordings for respiratory rate (OR: 62.5, 95% CI: 12.89 to 303.15); oxygen saturation (OR: 5.5, 95% CI: 1.05 to 28.95), level of consciousness (OR: 5.95, 95% CI: 1.62 to 21.84), and all seven parameters (OR: 20.1, 95% CI: 1.08 to 375.09, using Haldane’s estimator).

**Table 6:** **Numbers of patients with recordings and nurses’ responses to recordings that should have and did, or did not, trigger a response** ^**a**^ **in the first eight postoperative hours (per protocol analysis)**

	**Intervention arm**		**Control arm**				
**Parameter**	**MEWS chart n = 36 patients**		**Existing chart n = 57 patients**		***P*** **value**	**OR (df = 1)**	**95% CI**
	**Number (%)**	**Response triggered**	**Number (%)**	**Response triggered**			
Respiratory rate recorded	25 (69.4)		2 (3.5)		<0.001	62.50	12.89-303.15
Respiratory rate should have triggered a response	20 (55.6)	0	0	0	1.00		
Heart rate recorded	36 (100)		57 (100)		1.00		
Heart rate should have triggered a response	12 (33.3)	0	19 (33.3)	0	1.00		
Oxygen saturation recorded	6 (16.7)		2 (3.5)		0.03	5.5	1.05-28.95
Oxygen saturation should have triggered a response	1 (2.8)	0	1 (1.8)	0	1.00		
Systolic blood pressure recorded	36 (100)		57 (100)		1.00		
Systolic blood pressure should have triggered a response	15 (41.7)	3 (20.0)	25 (43.9)	1 (4.0)	0.23	5	0.48-52.53
Temperature recorded	35 (97.2)		54 (94.7)		1.00	1.94	0.19-19.45
Temperature should have triggered a response	17 (47.2)	0	22 (38.6)	0	1.00		
Level of consciousness recorded	33 (91.7)		37 (64.9)		0.004	5.95	1.62-21.84
Level of consciousness should have triggered a response	6 (16.7)	0	7 (12.3)	0	1.00		
Urine output recorded	33 (91.7)		51 (89.5)		0.12	1.29	0.30-5.54
Urine output should have triggered a response^b^	17 (47.2)	0	16 (28.1)	0	1.00		
All parameters recorded	5 (13.9)		0		0.003	20.08^b^	1.08-375.09^b^
Incomplete recording of all parameters	31 (86.1)		57 (100)				

For 21 patients in the intervention arm, the MEWS chart had not been used.

^a^MEWS trigger of 1 = recheck measurement after half an hour and report if no improvement; MEWS trigger of 2 = recheck measurement after five minutes and report immediately if no improvement; MEWS trigger of 3 = critical, report urgently.

^b^Haldane’s estimator was used for calculating OR (this circumvents zero values in cells by adding one half to each cell).

In the intervention wards nurses responded to 3.4% (three out of 88) MEWS that should have triggered reporting versus 1.1% (one out of 90) in the control wards (chi-squared = 1.07, df = 1, *P* = 0.30). Overall, nurses in both arms reported a combined total of four out of 178 (2.2%) deranged physiological parameters for four out of 93 (4.3%) patients who needed to be assessed (three in the intervention arm, and one in the control arm). CI, confidence interval; MEWS, modified early warning score; OR, odds ratio; df, the degrees of freedom, or the number of ‘entities’ that are free to vary when a statistical test is applied; this also determines the probability distribution used for the test statistic (see footnote to Table 3).

#### Number of times each vital sign was recorded in the first eight postoperative hours by trial arm

The number of recordings (Table 7) was significantly different between the intervention and control arms for respiratory rate (Z = −5.42, *P* <0.001), heart rate (Z = −2.09, *P* = 0.036), systolic blood pressure (Z = −3.03, *P* = 0.002), temperature (Z = −2.742, *P* = 0.006), and level of consciousness (Z = −4.44, *P* <0.001). However, the rates of documented ‘abnormal’ vital signs (by MEWS criteria) were similar, and the incidence of serious adverse events (SAEs) was low.

**Table 7:** **Total numbers of recordings of parameters by trial arm (intention-to-treat analysis)**

**Parameter**	**Number of recordings**			**Mean rank**	**Z value**	***P*** **value**
**Respiratory rate**	**Total number**	**Median**	**Range**			
Intervention arm (N = 57)	73	0	0	70.33	−5.42	<0.001
Control arm (n = 57)	2	0	0-2	44.67		
Heart rate						
Intervention arm (N = 57)	285	4	1-16	51.08	−2.09	0.036
Control arm (n = 57)	346	5	1-16	63.92		
Oxygen saturation						
Intervention arm (N = 57)	10	0	0-3	60.04	−1.75	0.080
Control arm (n = 57)	2	0	0-1	54.96		
Systolic blood pressure						
Intervention arm (N = 57)	325	6	1-18	48.24	−3.03	0.002
Control arm (n = 57)	414	7	1-19	66.76		
Temperature						
Intervention arm (N = 57)	134	2	1-10	65.69	−2.742	0.006
Control arm (n = 57)	113	2	0-7	49.31		
Level of consciousness						
Intervention arm (N = 57)	134	1	0-10	70.39	−4.44	<0.001
Control arm (n = 57)	38	1	0-2	44.61		
Urine output						
Intervention arm (N = 57)	93	2	0-6	58.60	0-.373	0.709
Control arm (n = 57)	87	1	0-4	56.40		

*P* values determined using the Mann-Whitney U test.

### Nurse participants

**Figure 2:**
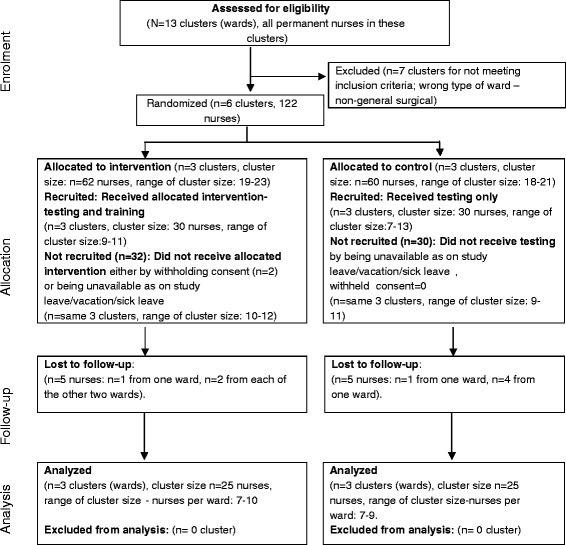
**Flow diagram of clusters and nurses for the intervention group (MEWS knowledge testing and training program) and control group (only knowledge testing).**

The flow of nurses recruited for the training intervention on an intention-to-treat basis is shown in Figure 2 [16-18]. It was anecdotally reported, after the trial conclusions, that a small number of two to three nurses from control wards were working occasional overtime shifts in intervention wards. A total of 50 nurses participated in pre- and post-knowledge testing (25 in each arm) (Additional file 3). There were 13 Registered Professional Nurses (RPNs) in each trial arm, five Registered Staff Nurses (RSNs) in the intervention arm versus four in the control arm, and seven Registered Nursing Auxiliaries (RNAs) in the intervention arm versus eight in the control arm. One nurse in each of two intervention wards declined to participate in the study.

#### Comparison of pre- and post-intervention knowledge scores within and between trial arms

The mean difference (four out of 23, 19.5%) between pre- (10 out of 23, 41.9%) and post-intervention scores (14 out of 23, 61.4%) in the intervention arm reached statistical significance (paired t-value 3.8, 95% CI: −30.0 to 8.9, *P* = 0.001) but not in the control arm (Table 8). The independent t-test result (Table 9) indicated that improvement in knowledge scores between trial arms was significantly better in the intervention arm than the control arm (mean difference: 15.5% (three out of 23), 95% CI: 3.8 to 27.2, *P* = 0.01, t-value: 2.69 (35.9 degrees of freedom), equal variances not assumed).

**Table 8:** **Comparison of pre- and post-intervention knowledge scores within each trial arm**

	**Pre-intervention score**	**Post-intervention score**	**Paired t-test for equality of means within trial arms**		
	**Mean score (%, SD)**	**Mean score (%, SD)**	**Mean difference (%, SD)**	**t-value (df)**	***P*** **value**
Intervention arm (n = 25)	10/23 (41.9, 14.6)	14/23 (61.4, 27.9)	4/23 (19.5, 25.6)	3.804 (24)	0.001
Control arm (n = 25)	9/23 (37.2, 18.19)	10/23 (41.2, 16.22)	1/23 (4.0, 13.2)	−1.512 (24)	0.144

**Table 9:** **Comparison of pre- and post-intervention knowledge scores between trial arms**

**Intervention arm (n = 25)**		**Control arm (n = 25)**		**Independent t-test for mean differences in pre- and post-intervention scores between trial arms**		
**Mean score difference (%, SD)**	**95% CI**	**Mean score difference (%, SD)**	**95% CI**	**Mean score difference (%, 95% CI)**	**t-value (df)**	***P*** **value**
4/23 (19.5, 25.6)	8.9-30.0	1/23 (4.0, 13.2)	−1.5-9.5	3/23 (15.5, 3.8-27.2)	2.69 (35.9) equal variances not assumed	0.011

Data were normally distributed and parametric tests were used.

The 95% CI values with minus signs indicate a wide standard deviation within the greater population of nurses, particularly in the control wards, indicating that the sample size may be too small.

Note: test material is available to reviewers and editors on request. CI, confidence interval, SD, standard deviation; df, degrees of freedom.

## Discussion

To our knowledge, this is the first report of a randomized controlled trial of a MEWS. A pragmatic, cluster randomized, parallel group, controlled clinical trial of a training program and a MEWS chart for the detection of early signs of clinical deterioration made significant differences to knowledge, recording of respiratory rate, and recording of all seven physiological parameters during the first eight postoperative hours. However, there was little evidence that the intervention improved nurses’ responses to patients who triggered a critical MEWS reporting algorithm.

### Limitations

Difficulties with the conduct, analysis, and reporting of cluster trials have been reported [36] in both developed countries and sub-Saharan Africa [37,38]. Approval for implementation and testing of the MEWS in this study was limited to the period of study and the researchers had no authority to plan for its further use in practice thereafter. Although a cluster trial is intended to minimize contamination, professionals in the control arm may be sensitized to the problems under investigation by their involvement with researchers or communication with colleagues in the intervention arm [39]. Any nurses from control wards, untrained in the MEWS, voluntarily working overtime shifts in intervention wards might have diluted the impact of the intervention [40]; this may account for some, but not all, of the non-use of 37% of MEWS charts and the improvement in nurses’ knowledge in control wards. Unusually for a randomized controlled trial, this work was conducted in a real-world setting in a resource-poor environment in a developing country. As in most cluster trials, we were unable to link individual nurses and patients, and most patients would have received care from several nurses. MEWS charts are not problem-free [41], and the complexity of introducing a EWS system and educational program suggest that pragmatic cluster randomized controlled trials or stepped wedge trials might be preferable [42] to patient randomized controlled trials [43-45].

Suboptimal compliance with the MEWS affected the trial; no scores were totaled. Complex systems such as EWS are prone to human calculation errors, when ‘pen and paper’ is used [45], rather than electronic systems [46]. Not all patients in both trial arms had recordings of all seven vital signs. In our previous work [13] in the same six wards, results showed a similar pattern of recording of vital signs, and no patients’ records contained recordings for all seven parameters displayed on the MEWS. The absence of a total MEWS or a MEWS for one or more (of six) vital signs in 47% (n = 509) of patients in general surgical and medical wards in an observational data analysis pre- and post-intervention study in Belgium [47] resonates with our findings. There were differences in recordings between trial arms but similar rates of ‘abnormal’ vital signs (by MEWS criteria). The absence of catastrophic clinical consequences in both trial arms is noteworthy, but not unusual in our clinical areas [47], and we have no evidence that the MEWS improved clinical outcomes.

Our previous work [13] in the same wards indicated a similar uniform absence of responses to abnormal vital signs. Per protocol analyses indicated that, where used, the MEWS increased recordings of oxygen saturation and level of consciousness, in addition to respiratory rate. In both intention-to-treat and per protocol analyses, more patients in the intervention arm had abnormalities reported. However, differences did not reach statistical significance. Those that comply with trial protocols are not a random sample and may be different in some ways. Selectively excluding those who violate protocols may introduce bias, necessitating cautious interpretation of per protocol analyses. We acknowledge a paucity of conservatism in our sample size calculation, most particularly that we were unable to take account of clustering; however, data collection was limited by available resources. A larger sample is needed to test these findings. Our findings are presented without correction for multiple testing [48,49] in order to delineate leads for larger trials, and interpretation rests with readers.

Traditionally, nursing work that is not recorded is regarded as incomplete or even ‘not done’. However, the absence of recorded interventions for patients meeting pre-determined criteria for intervention, although of deep concern, does not mean necessarily that there were no interventions. Nevertheless, without a documented record, nurses’ reporting is incomplete, and counted as such in this study. From single site research we cannot assume that respondents and response patterns are representative of other populations. The trial location may have influenced recruitment, retention, and clinical outcomes. A new vital signs chart may improve recording due to increased interest [9,50].

No nurse observation protocol existed at our research site. Intervals between manual monitoring of vital signs on general wards vary substantially, for example between eight and 12 hours [51]. Our study might have been strengthened by implementing and evaluating compliance with a standard protocol for frequency of physiological observations of at least every 12 hours for every patient, as recommended in the United Kingdom’s National Institute for Health and Care Excellence (NICE) Clinical Practice Guideline 50 [52]. There were few deaths and too many confounding factors in a clinical setting to attribute these outcomes to inadequate recording and reporting. A much larger sample would be needed to examine the effect of the MEWS on SAEs.

### The realities of practice

Incomplete recording of all seven vital signs in the postoperative period was high in the intervention arm and worse in the control arm. In the control arm, poor recording might be attributed to the absence of guidelines for appropriate frequency [9] and number of vital signs to be recorded. Not all MEWS charts include monitoring of urine output [9]. Poor recording of oxygen saturation following the administration of a general anaesthetic and opioids [53] is of concern.

The improvement in scores between pre- and post-intervention knowledge tests of nurses in the intervention arm from 41.9 to 61.4% did not translate into improved documented reporting of patients who met the pre-determined criteria for intervention. The MEWS does not replace clinical judgment in detecting deteriorating patients [21]. Even so, nurses failed to report deranged physiological parameters for 87.7% (intervention) and 96.5% (control) patients. This is deeply concerning and raises questions about the specificity of the MEWS to identify ‘at risk’ patients. However, in our previous work [13] in the same wards, there were no reported interventions for 10 out of 11 (90.9%) patients who died and had abnormal physiology, and for 38 (86.4%) control patients with abnormal physiology. The specificity of the Cape Town MEWS had been determined at between 77.3 and 81.4% [21]. Other studies also report nurses’ failure to communicate concerns and inappropriate responses when patients showed signs of physiological deterioration [3,9,54]. There are too many confounding factors in a clinical setting to attribute these outcomes to inadequate recording and reporting. Strategies for implementing and monitoring patient safety initiatives in large tertiary hospitals need to accommodate the complexity of such organizations. Patients admitted to this level of care have complex conditions requiring treatment not available at secondary level hospitals, increasing their risk of adverse events. Other factors have to be considered.

Problems arise when nurses are competent in using technology for the monitoring of vital sign but lack clinical knowledge in interpreting data and intervening appropriately to ensure optimum and safe patient care. Misinterpretation of clinical data is associated with poor clinical reasoning skills and, in studies using computer-based clinical scenarios, nurses have been found to overestimate risks and the need to intervene [55]. An Australian survey found that nurses may not always follow predetermined medical emergency team (MET) calling criteria and may not recognize when assistance is required [56].

Poor recording of vital signs and little evidence of appropriate responses to signs of physiological deterioration may be linked to ‘task allocation’, the method of patient care employed in the research wards, in preference to ‘patient allocation’. It is standard practice in South African wards to delegate monitoring, recording, and interpretation of patient observations to nursing auxiliaries and enrolled nurses, and the quality of recording should be viewed within this context. The core function of the nurse in avoiding SAEs should go beyond the recording of patients’ physiological vital signs [57]. It is the nurse’s professional responsibility to understand the significance of patient observations [58,59].

### Benefits of the intervention

Disappointingly, the improved knowledge scores only reached 61.4%, and may have been impacted by language difficulties and the inclusion of non-professional nurses who have a non-academic qualification. The MEWS chart resulted in significantly more patients in the intervention wards than control wards having recordings of respiratory rate, the best discriminator for clinical outcomes [4]. Not all existing observation charts [12] included respiratory rate monitoring. This trial showed that, if included, respiratory rate is more likely to be monitored. As respiratory rate monitoring is the best discriminator of SAEs, it has replaced recording of oxygen saturation on some charts [9]. Nevertheless, the improved scores suggest that a hospital-wide training program for early recognition and management of patients with impending critical illness is needed, in order to complement in-service education programs for late rescue techniques, such as cardiopulmonary resuscitation (CPR). Low pre-intervention bioscience knowledge scores in both trial arms confirm previous research [7] that suggests that this underpinning knowledge may be suboptimal.

Improved recording did not translate into an improvement in responses to deranged physiology. The Cape Town ward MEWS has a wider range of cut points (thresholds) for respiratory rate, oxygen saturation, heart rate, systolic BP, and temperature than other published MEWS. This might have made interpretation of parameter readings more difficult, and may have accounted for the low response rate to the large number of triggers, despite training. This raises questions regarding the clinical effectiveness of the MEWS chart deployed to identify patients at risk and the user friendliness of the chart. To address the latter possibility, a revised version of the Cape Town ward MEWS observations chart (Additional file 4: Figure S2: Revised Cape Town MEWS chart) is presented. The United Kingdom National Early Warning Score (NEWS) [60] was not available when the study was undertaken. Since completion of this trial, we have substituted the values from the NEWS into the Cape Town ward MEWS, with permission from the United Kingdom Royal College of Physicians, as the NEWS values retain the range of trigger cut points (thresholds) (heart rate: 111 to 129; systolic BP: 81 to 100) we found to be associated with mortality [21]. User friendliness of the chart might be improved by reducing the number of cut points (thresholds) for physiological parameters that were not associated with mortality. To assist those responding to patients’ physiological deterioration, the United Kingdom NEWS algorithm for a total MEWS [60] has been included on the revised chart, in addition to guidelines for reporting a single parameter trigger.

The association between recordings of vital sign parameters (including respiratory rates) and mortality [61-63] challenges traditional assumptions that mortality outcomes and determinants of survival fall solely within the domain of medical care, and provides further evidence that these outcomes are ‘nursing sensitive’ [8]. Nurses are concerned that patients on general wards are having increasingly more complex surgery, increasing their dependence and morbidity which, in the face of understaffing, results in increased workload and suboptimal quality of care, leaving less time to apply learning in practice [64]. Specialist nurses, increased registered nurse (RN)-to-patient ratios and a richer RN skill mix (more RNs than other categories of nurses), is inversely related to hospital mortality rates (n = 10,184 nurses in 168 hospitals) [65] and to most adverse events (n = 124,204 patients) [66]. These results should be generalized cautiously as hospital characteristics vary [67]. A total of 19 of 27 studies that were systematically reviewed found an association between one or more unfavorable nursing environmental attributes and higher mortality. Despite extensive variability in attribute and outcome measures, settings, and research quality across studies, there is evidence that social and environmental characteristics of hospital nursing practice affect the outcomes of care, but more research is needed to link the nursing environment, including patient monitoring, to patient outcomes [68].

## Conclusions

The monitoring of vital signs should be central to patient safety initiatives and hospital-wide morbidity and mortality reviews. Recording and analyzing trends in the monitoring of vital signs may alert managers to changing standards of care. The Cape Town MEWS observations chart as a combined (aggregated and single parameter) track-and-trigger tool [12] has been revised to indicate more clearly when abnormalities should be reported, and requires further testing. We recommend improved training for appropriate reporting of deranged physiology.

## Additional files

Additional file 1: Figure S1. Consensus derived Cape Town MEWS observations chart and reporting algorithm. Additional file 2:
**CONSORT Statement extended to pragmatic cluster trials: checklist.**
Additional file 3:
**Pre- and post-intervention test.**
Additional file 4: Figure S2.
**Revised Cape Town MEWS chart.**

## Abbreviations

ALERT: Acute Life-threatening Events - Recognition and Treatment
BP: Blood pressure
CI: Confidence Interval
CPR: Cardiopulmonary Resuscitation
EWS system: Early warning scoring system
EWS: Early warning score
HDU: High dependency unit
HREC: Human Research Ethics Committee
ICU: Intensive Care Unit
MEWS system: Modified early warning scoring system
MEWS: Modified early warning score
NEWS: National Early Warning Score
NICE: National Institute for Health and Care Excellence
NHREC: National Health Research Ethics Committee
OR: Odds Ratio
RE: Risk Estimate
RN: Registered Nurse
RPN: Registered Professional Nurse
RSN: Registered Staff Nurse
RNA: Registered Nursing Auxiliary
SAE: Serious adverse event
SPI: Safer Patients Initiative
