# Supplementary figures and images for: Early warning scoring systems versus standard observations charts for wards in South Africa: a cluster randomized controlled trial
Source: Trials. 2015 Mar 20;16:103. doi: 10.1186/s13063-015-0624-2 (PMC4374204; doi:10.1186/s13063-015-0624-2)

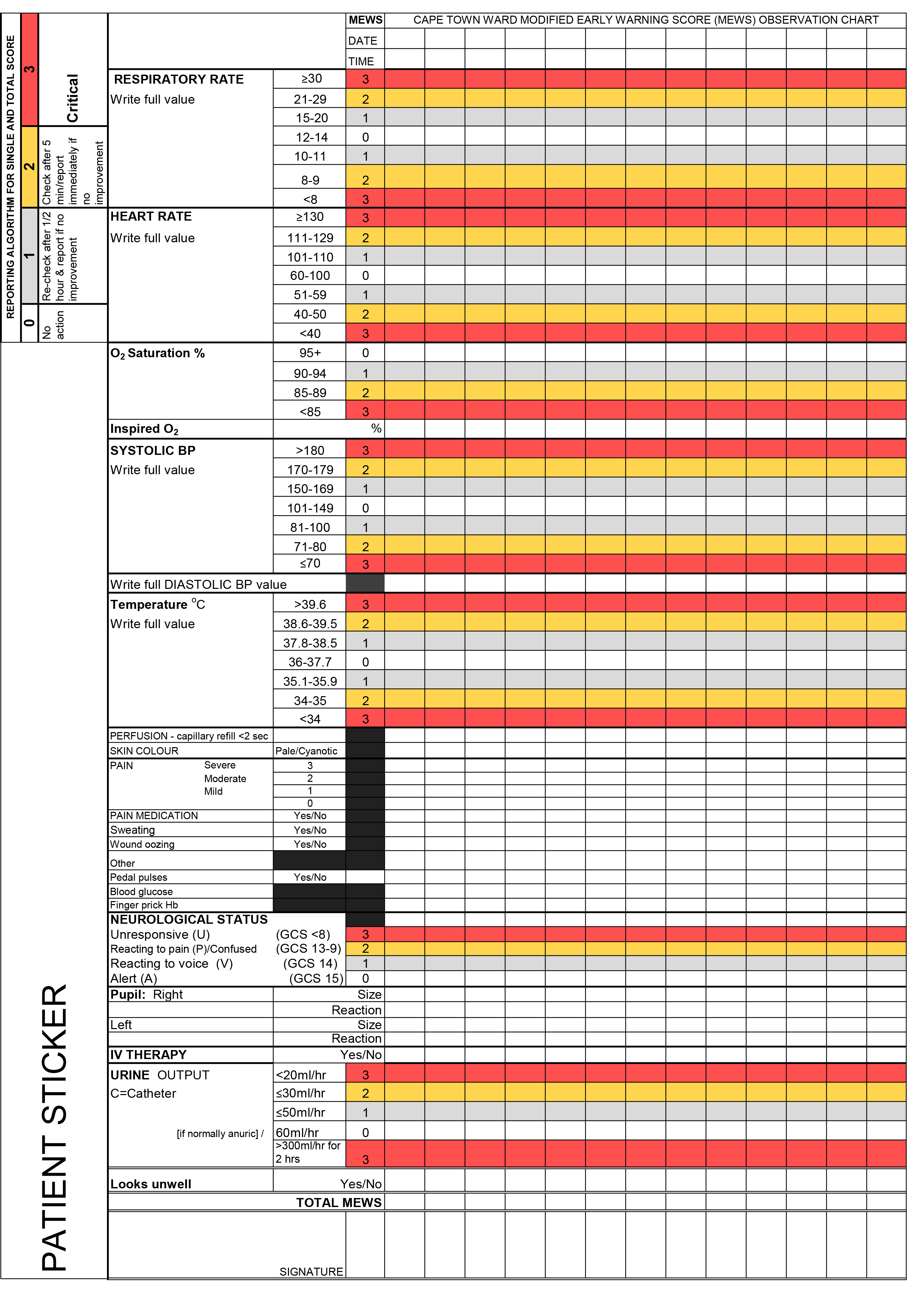

Supplement: Additional file 1: Figure S1. — Consensus derived Cape Town MEWS observations chart and reporting algorithm. [file 13063_2015_624_MOESM1_ESM.tiff]
