# Supplementary material for: Early warning scoring systems versus standard observations charts for wards in South Africa: a cluster randomized controlled trial
Source: Trials. 2015 Mar 20;16:103. doi: 10.1186/s13063-015-0624-2 (PMC4374204; doi:10.1186/s13063-015-0624-2)
Supplement: Additional file 2: — CONSORT Statement extended to pragmatic cluster trials: checklist. [file 13063_2015_624_MOESM2_ESM.docx]

Additional file 2

CONSORT Statement extended to pragmatic cluster trials: checklist

| Section | Item | Standard CONSORT description | Extension for pragmatic *cluster* trials | Application to Study Section in paper |
| --- | --- | --- | --- | --- |
| TITLE AND ABSTRACT | | | | |
| Design | 1 | How participants were allocated to interventions (eg, “random allocation,” “randomised,” or “randomly assigned”) | *specifying that allocation was based on clusters.* Describe the health or health service problems that the intervention is intended to address and other interventions that may commonly be aimed at this problem | Methods p. 5 |
| INTRODUCTION |  |  |  |  |
| Background | 2 | Scientific background and explanation of rationale | *including the rationale for using a cluster design* | p. 4 |
| METHODS |  |  |  |  |
| Participants | 3 | Eligibility criteria for participants; settings and locations where the data were collected | Eligibility criteria should be explicitly framed to show the degree to which they include typical participants and/or, where applicable, typical providers (eg, nurses), institutions (eg, hospitals), communities (or localities eg, towns) and settings of care (eg, different healthcare financing systems) | Sampling p. 5-6  Fig 1, Fig 2 |
| Interventions | 4 | Precise details of the interventions intended for each group and how and when they were actually administered | *whether they pertain to the individual level, the cluster level, or both*  Describe extra resources added to (or resources removed from) usual settings in order to implement intervention.  Indicate if efforts were made to standardise the intervention or if the intervention and its delivery were allowed to vary between participants, practitioners, or study sites  Describe the comparator in similar detail to the intervention | Interventions p. 8-9 |
| Objectives | 5 | Specific objectives | *and whether they pertain to the individual level, the cluster level, or both* | Abstract p. 2 Individual and cluster |
| Outcomes | 6 | Clearly defined primary and secondary outcome measures and, when applicable, any methods used to enhance the quality of measurements (eg, multiple observations, training of assessors) | *whether they pertain to the individual level, the cluster level, or both.*  Explain why the chosen outcomes and, when relevant, the length of follow-up are considered important to those who will use the results of the trial | Abstract p. 2  Methods p. 9 |
| Sample size | 7 | How sample size was determined; explanation of any interim analyses and stopping rules when applicable | *including method of calculation, number of clusters, cluster size, a coefficient of intracluster correlation (ICC or k), and an indication of its uncertainty*  If calculated using the smallest difference considered important by the target decision maker audience (the minimally important difference) then report where this difference was obtained | Methods p. 5-6  This calculation took no account of clusters, and the low number of patients with all vital signs recorded in the preparatory work precluded calculation of ICC at this stage. |
| RANDOMISATION |  |  |  | p.7 |
| Sequence generation | 8 | Method used to generate the random allocation sequence, including details of any restriction (eg, blocking, stratification) | *or matching* | Methods p. 7 |
| Allocation concealment | 9 | Method used to implement the random allocation sequence (eg, numbered containers or central telephone), clarifying whether the sequence was concealed until interventions were assigned | *specifying that allocation was based on clusters rather than individuals* | Methods p. 7 |
| Implementation | 10 | Who generated the allocation sequence, who enrolled participants, and who assigned participants to their groups |  | Methods p. 7 |
| Blinding (masking) | 11 | Whether participants, those administering the interventions, and those assessing the outcomes were blinded to group assignment | If blinding was not done, or was not possible, explain why | Methods p. 7-8 |
| Statistical methods | 12 | Statistical methods used to compare groups for primary outcome(s); methods for additional analyses, such as subgroup analyses and adjusted analyses | *indicating how clustering was taken into account* | Data analysis p.10  Due to low numbers in some cells, there were no adjusted analyses |
| RESULTS |  |  |  | p. 10 |
| Participant flow | 13 | Flow of participants through each stage (a diagram is strongly recommended)—specifically, for each group, report the numbers of participants randomly assigned, receiving intended treatment, completing the study protocol, and analysed for the primary outcome; describe deviations from planned study protocol, together with reasons | The number of participants or units approached to take part in the trial, the number which were eligible, and reasons for non-participation should be reported | p. 10 & Figure 1  p. 13 & Fig 2 |
| Recruitment | 14 | Dates defining the periods of recruitment and follow-up |  | Study sites p. 5, 6, 7 |
| Baseline data | 15 | Baseline demographic and clinical characteristics of each group | *for the individual and cluster levels as applicable* | Records p. 11-13,  Tables 1-6 p. 25-30  Nurses p. 13,  Table 7 p.31 |
| Numbers analysed | 16 | Number of participants (denominator) in each group included in each analysis and whether analysis was by “intention-to-treat”; state the results in absolute numbers when feasible (eg, 10/20, not 50%) |  | Tables 1-8 p. 24-31  Table 6 (ITT) p. 29  Table 7 (per protocol) p. 30 |
| Outcomes and estimation | 17 | For each primary and secondary outcome, a summary of results for each group and the estimated effect size and its precision (eg, 95% CI) | *and a coefficient of intracluster correlation (ICC or k) for each primary outcome*. | Tables 1-8 p. 24-31  Table 6 (ITT) p. 29  Table 7 (per protocol) p. 30 |
| Ancillary analyses | 18 | Address multiplicity by reporting any other analyses performed, including subgroup analyses and adjusted analyses, indicating which are prespecified and which are exploratory |  | Ancillary analyses are reported in Kyriacos (2011, thesis_ |
| Adverse events | 19 | All important adverse events or side effects in each intervention group |  | No adverse events |
| DISCUSSION |  |  |  |  |
| Interpretation | 20 | Interpretation of the results, taking into account study hypotheses, sources of potential bias or imprecision, and the dangers associated with multiplicity of analyses and outcomes |  | p. 13-19 |
| Generalisability | 21 | Generalisability (external validity) of the trial findings | *to individuals and/or clusters (as relevant)*  Describe key aspects of the setting which determined the trial results. Discuss possible differences in other settings where clinical traditions, health service organisation, staffing, or resources may vary from those of the trial | Limitations of the study p. 15 |
| Overall evidence | 22 | General interpretation of the results in the context of current evidence |  | Discussion p. 13-19 |

(Campbell MK, Elbourne DR, Altman DG. CONSORT statement: extention to cluster randomized trials. British Medical Journal 2008; 328: 703. Zwarenstein M, Treweek S, Gagnier JJ, Altman DG, Tunis S, Haynes B, Oxman AD, Moher D, 2008).
