# Supplementary material for: Early warning scoring systems versus standard observations charts for wards in South Africa: a cluster randomized controlled trial
Source: Trials. 2015 Mar 20;16:103. doi: 10.1186/s13063-015-0624-2 (PMC4374204; doi:10.1186/s13063-015-0624-2)
Supplement: Additional file 3: — Pre- and post-intervention test. [file 13063_2015_624_MOESM3_ESM.zip › 13063_2015_624_add3.rtf]

Additional file 3
PRE- and POST-INTERVENTION TEST 
Title of study: The development, validation and testing of a vital signs monitoring tool for early identification of deterioration in adult surgical patients
Please answer all the questions on this sheet: 
Office use
	1	
1.	How would you recognize respiratory arrest? (one sign)
1.1
	2	
2.	There is a sudden change in a patients' condition: circle the 2 respiratory rate readings in the list below for which you will summon more skilled assistance (help):  in other words draw a circle around one group of slow rate readings and one group of fast rate readings.
Less than 8	8-9	10-11	12-14	15-20	21-29	30 or more	
	3	
3.	How would you recognize signs of inadequate breathing in a patient in your ward? Give 3 signs.
3.1
3.2
3.3
4.	Do you personally measure and record oxygen saturation (SAT/SpO2) on your ward? Circle your answer. Yes		No
5.	There is a sudden change in a patients' condition: circle the one SAT/SpO2 reading in the list below for which you will summon more skilled assistance (help): in other words draw a circle around one group of readings.
Less than 85%	85-89%	90-94%	95+%			
1

	
6.	List 3 common causes of breathlessness (shortness of breath) in a post-operative patient: 
6.1
	3	
6.2
6.3
	1	
7.	How would you recognize cardiac arrest? (one sign)
7.1
	2	
8.	There is a sudden change in a patients' condition: circle the 2 heart rate values in the list below for which you will summon more skilled assistance (help): in other words draw a circle around one group of slow rate readings and one group of fast rate readings.
Less than 40 bpm	40-50	51-59	60-100	101-110	111-129	130 or more	
	2	
9.	There is a sudden change in a patients' condition: circle the 2 systolic blood pressure values in the list below for which you will summon more skilled assistance (help): in other words draw a circle around one group of low readings and one group of high readings.
70 or less	71-80	81-100	101-149	150-169	170-179	180 or more	

	3	
10.	List 3 causes of low blood pressure: 
10.1
10.2
10.3
	3	
11.	List 3 causes of high blood pressure: 
11.1
11.2
11.3
12.	List 4 factors responsible for maintaining a normal blood pressure (how does the body maintain a normal blood pressure)? 
	4	
12.1
12.2
12.3
12.4
	5	
13.	 List 5 factors that could help you assess cardiac output clinically [in other words, by looking at or examining the patient without using equipment, how would you know that the heart was pumping adequately]? 
13.1
13.2
13.3
13.4
13.5
	1	
14.	 Circle the one group of temperature readings in the list below for which you will take no action:
Less than 34o C	34-35	35.1-35.9	36-37.7	37.8-38.5	38.6-39.5	39.6 or more	
	1	
15.	 Circle one response in the list below for a sudden deterioration in a patients' conscious level that will alert you to call for more skilled assistance (in other words, when will you call for help?):
ALERT (A)

(same as GCS 15)	RESPONDS TO VOICE (V)

(same as GCS 14)	RESPONDS TO PAIN (P) / Confused
(same as GCS 13-9)	UNRESPONSIVE (U)

(same as GCS <8)	
	2	
16.	Circle the 2 values for urine output in the list below for which you would seek more skilled assistance (help):
20 ml/hr or less	30 ml/hr or less	50 ml/hr or less	60 ml/hr
			>300 ml/hr for 2 hrs	

	34	


Note: Questions 1, 3, 6, 7, 10, 11, 12, 13 accounted for the 23 mark knowledge test.
Staggered pre-intervention knowledge testing was conducted between 12 March and 23 March for nurses in both trial arms. The MEWS training programme was conducted between 12 March and 22 April. The MEWS chart was introduced on 1 May and removed at midnight on 31 July. Post-intervention knowledge testing was conducted between 3 August and 13 August.
