# Supplementary material for: Early warning scoring systems versus standard observations charts for wards in South Africa: a cluster randomized controlled trial
Source: Trials. 2015 Mar 20;16:103. doi: 10.1186/s13063-015-0624-2 (PMC4374204; doi:10.1186/s13063-015-0624-2)
Supplement: Additional file 4: Figure S2. — Revised Cape Town MEWS chart. [file 13063_2015_624_MOESM4_ESM.pdf]

MODIFIED EARLY WARNING SCORING (MEWS) VITAL SIGNS CHART

SCORING KEY for single MEWS

0	1	2	3
No action	Re-check after 1/2 hour/report if no improvement	Check after 5 min/report immediately if no improvement	Single RED score of 3 = Medium risk but still report immediately

SCORING KEY for TOTAL MEWS

0, Total 1-4 = Low risk Re-check after 1/2 hr /report if no improvement	Total 5-6 = Medium risk Check after 5 min/report immediately if no improvement	Total score 7 or more = High risk Report immediately

PATIENTS' NORMAL BP

PATIENT'S IDENTIFICATION & HOSPITAL NUMBER STICKER

		SCORE										SCORE	
	POST-OPERATIVE DAY												
	DATE												
	TIME												
**RESPIRATORY RATE**	25 or more	3										3	25 or more
Write in full if outside the range eg. 7 or 26	21-24	2										2	21-24
	12-20	0										0	12-20
	9-11	1										1	9-11
	≤8	3										3	≤8
**O_2 Saturation %**	96+	0										0	96+
	94-95	1										1	94-95
	92-93	2										2	92-93
	91 or less	3										3	91 or less
**Inspired O_2**	YES % / NO												YES % / NO
**Temperature ^{o}C**	39.1 or higher	2										2	39.1 or higher
Write in full if outside the range eg.	38.1-39.0	1										1	38.1-39.0
	36.1-38.0	0										0	36.1-38.0
	35.1-36.0	1										1	35.1-36.0
	35 or lower	3										3	35 or lower
**HEART RATE**	131 or more	3										3	131 or more
Write in full if outside the range eg. 39 or 132	111-130	**2**										**2**	111-130
	91-110	1										1	91-110
	51-90	0										0	51-90
	41-50	1										1	41-50
	40 or less	3										3	40 or less
**SYSTOLIC BP**	220 or more	3										3	220 or more
Write in full if outside the range eg. 90 or 221	111-219	0										0	111-219
	101-110	1										1	101-110
	91-100	2										2	91-100
	90 or less	3										3	90 or less
**DIASTOLIC BP** write in full eg. 80													
PERFUSION - capillary refill <2 sec													Perfusion
SKIN COLOUR	Pale/Cyanotic												Pale/Cyanotic
PAIN Severe	3												Pain 3
Moderate	2												2
Mild	1												1
No pain	0												0
HAD PAIN MEDICATION	YES/NO												YES/NO
Sweating	YES/NO												YES/NO
Wound oozing	YES/NO												YES/NO
Other: write													
Pedal pulses	YES/NO												YES/NO
Blood glucose													
Finger prick Hb													
**LEVEL OF CONSCIOUSNESS**													
Alert (A)	(GCS 15)	0										0	A
Reacting to voice (V) / Pain (P) / Unresponsive (U)	(GCS 14 or less)	3										3	V/P/U
**Pupil size:**													
Right	Size												Right: Size
	Reaction												Reaction
Left	Size												Left: Size
	Reaction												Reaction
**Intravenous fluid**	YES/NO												IV YES/NO
**URINE** OUTPUT	more than 300ml/hr for 2	1										1	more than 300ml/hr for 2
[Indicate if normally anuric]	60ml/hr	0										0	60ml/hr
C=Catheter	less than 60ml/hr	1										1	less than 60ml/hr
	less than 30ml/hr	2										2	less than 30ml/hr
	**No output**	3										3	**No output**
**Looks unwell**	YES/NO												YES/NO
	**TOTAL SCORE**												**TOTAL SCORE**
	Initials												

This document by Una Kyriacos (Ph.D. RN, UCT Division of Nursing & Midwifery, 2014) is licensed under the Creative Commons Attribution-NoDerivatives 4.0 International Licence. The licence can be viewed at http://creativecommons.org/licenses/by-nd/4.0/.
This work was inspired by the Royal College of Physicians' National Early Warning Score (NEWS) 2012.
